# Disaster risk index on disaster management budgeting: Indonesia’s national data set

**DOI:** 10.4102/jamba.v15i1.1365

**Published:** 2023-02-17

**Authors:** Nurhayati Haris, Andi C. Furqan, Abdul Kahar, Fikry Karim

**Affiliations:** 1 Department of Accountancy, Faculty of Economics and Business, Universitas Tadulako, Palu, Indonesia; 2 Center For Sustainable Governance Studies, Faculty of Economics and Business, Universitas Tadulako, Palu, Indonesia

**Keywords:** Disaster risk index, disaster response emergency fund, fiscal budgeting, disaster mitigation, Indonesia

## Abstract

This study analysed Indonesian local government budgeting model based on the Disaster Risk Index (DRI). This study used a sample of local governments in Indonesia consisting of provincial, regency and municipality levels, especially for 2015–2019 data with a final sample of 2609 observations. The results of the analysis and testing showed that most of the Indonesian local governments fall into the high category in the DRI. The DRI has a positive effect on the Disaster Response Emergency Fund (DREF). The results were robust to the differences in DRI measurements, both using scores and DRI categories. This study also found that the DRI has been used as the basis for budgeting regional expenditures. The budget was allocated in disaster-related public procurements such as public service, housing, public facilities and public health. The budgeting for the implementation of economic and social functions was not influenced by the DRI. Instead, the DRI was found to have a negative effect on the implementation of environmental functions. The findings showed that in general, DRI has been used as the basis for budgeting for regional disaster management, but it is still limited to functions related to disaster emergency response. The budgeting of functions related to the prevention stage has not been optimally carried out, especially by mitigating natural hazards through strengthening the quality of the environment.

**Contribution:**

The results are expected to contribute to the local government to improve disaster resilience through strengthening regional financial funding.

## Introduction

Indonesia is one of the countries with the highest natural hazard vulnerability in the world (Djalante et al. 2017). Supported by its tropical geographical conditions in the Pacific ring of fire and the Indo-Australian tectonic plate, the country is very vulnerable to geological and hydrometeorological natural hazards. From a geological point of view, some of the most frequent common disasters are earthquakes, volcanic eruptions and tsunamis, while from a hydrometeorological point of view, Indonesia faces storms, droughts, landslides, floods and abrasion in coastal areas (Suprapto et al. 2015). With the complexity of natural hazards, the *Badan Nasional Penanggulangan Bencana* (BNPB) [National Disaster Management Agency], as the highest authority in coordinating disaster management in Indonesia, classifies disaster hazards into three categories, namely low, medium and high. Furthermore, Suprapto et al. (2015) found that 97% of Indonesia’s population lives in disaster-prone areas, with earthquakes being the most dangerous to 62.4% of the population.

In addition, other disasters that need to be mitigated besides natural hazards are man-made hazards. According to the United Nations International Strategy for Disaster Reduction (UN-ISDR), disasters can be grouped into geological hazards, hydrometeorological hazards, biological hazards, technological hazards and environmental degradation. These high vulnerabilities are compounded by the lack of awareness and anticipation of the community towards disasters, as well as poor infrastructure, especially for evacuating, in cities or areas with high disaster risk.

However, this vulnerability is not balanced with coordinated and professional disaster management. In general, the handling mechanism is carried out on a temporary basis and is not sustainable. Also, it does not involve the grassroots or general population, which is a critical point in disaster management and mitigation. The lack of seriousness in handling disasters in Indonesia can be seen from the absence of standard operating procedures (SOPs) that are followed in the event of a disaster from the central to the regional levels. Furthermore, the thing that has become a concern in disaster management so far in Indonesia is that there is no visible, good coordination between technical institutions that handle natural hazards in Indonesia. For example, from the aspect of hydrogeographic disaster mitigation, it is not clear how coordination is carried out between the National Disaster Management Agency (BNPB) and the Meteorology, Climatology and Geophysics Agency (BMKG), as well as with relevant ministries (Kodoatie & Sjarief 2006). This shows that the development of disaster mitigation has not been carried out optimally either by the central government or local governments. In fact, various previous studies have shown that disaster mitigation capability is the right step to reduce disaster risk, both through physical development and increasing the ability to deal with disaster threats (Al-Nammari & Alzaghal 2015; Mulyana et al. 2022; Oh & Lee 2020).

Previous research arguably paid limited attention to the effect of the disaster risk index (DRI) on local government budgets. Data sourced from the Fiscal Policy Agency of the Ministry of Finance (2018) showed that between 2000 and 2016, the average direct economic loss in the form of damage to buildings and nonbuildings because of natural hazards in Indonesia reaches around IDR 22.8 trillion annually. One of disastrous events was the earthquake and tsunami in Aceh in 2004, which caused losses of up to IDR51.4 trillion, with the recovery process taking more than 5 years. The calculation of these losses does not include losses caused by 2564 disasters in 2018, including the earthquake in West Nusa Tenggara; the earthquake, liquefaction and tsunami in Central Sulawesi; and the tsunami in the Sunda Strait, with a total loss value of around IDR100 trillion (Asmara 2018) (see Table 1).

**TABLE 1:** Average losses due to disasters in Indonesia from 2002 to 2016.

Year	Amount
2002	143
2003	403
2004	775
2005	599
2006	740
2007	816
2008	1.073
2009	1.246
2010	1.941
2011	1.633
2012	1.811
2013	1.674
2014	1.967
2015	1.732
2016	1.062

*Source*: Jatim Newsroom, 2017, ‘Rata-Rata Kerugian Akibat Bencana Tiap Tahun Capai Rp 30 Triliun’, *Jatim Newsroom*, 26 April, Department of Communication and Information, East Java Province, viewed 11 September 2022, from https://kominfo.jatimprov.go.id/read/umum/rata-rata-kerugian-akibat-bencana-tiap-tahun-capairp-30-triliun

The magnitude of the potential for disasters in Indonesia and the resulting losses have not been followed by budgetary policies that support the strengthening of disaster management efforts. On average, the government provides disaster reserve funds of only IDR3.1 trillion per year. When compared with the average loss each year, there is a difference of around IDR19.7 trillion, whose funding has the potential to have a negative impact on the state’s fiscal condition. Since 2018, the national government has had a Disaster Risk Financing and Insurance (DRFI). It is the government’s priority program to deal with fiscal risks because of disasters. However, almost all local governments have not implemented this strategy. The magnitude of the socio-economic impact and the high uncertainty because of disaster has made several budget changes. The local government also has to refocus activities and reallocate the budget several times. The general objective of the study is to develop a local government budgeting model based on the DRI.

The Center for Disaster Data, Information and Communication of BNPB (2021) stated that Disaster Risk Assessment is carried out by calculating the components of hazard, vulnerability and capacity in each province and district or city. Hazard components are natural phenomena that can cause disasters such as earthquakes, volcanic eruptions, tsunamis, floods and others. The vulnerability component is the physical, sociocultural, economic and environmental conditions that are vulnerable to being exposed to disasters (Permana et al. 2021). Meanwhile, the capacity component consists of elements of regional resilience such as policies and institutions, education and training, logistics, mitigation capacity, prevention, preparedness and emergency response and recovery capacity.

The results of the calculation of the DRI in 2021 show that 15 provinces are 7 in the high disaster risk and 19 provinces are in the medium disaster risk and no province is in the low disaster risk. The three provinces with the highest risk are West Sulawesi (score 164.85), Bangka Belitung Islands (160.98) and Maluku (160.84). Meanwhile, the three provinces that have the lowest medium risk are West Nusa Tenggara (122.33), Riau Islands (114.71) and Jakarta (60.43). Of the 514 regencies or cities in Indonesia, there are 221 urban districts that are in the high risk and 293 that are in the medium risk categories. The three districts or cities with the highest scores are Southwest Maluku, Maluku province (score 223.20); Majene, West Sulawesi province (score 217.62); and South Halmahera, North Maluku province (score 216.99). Meanwhile, the three with the lowest medium scores are South Jakarta (49.89), Seribu Islands (49.46) and Central Mamberamo (44.80) (Center for Disaster Data, Information and Communication of BNPB 2021).

The DRI provides an overview of the achievements of disaster management efforts at the provincial and district or city levels. The values listed are the results of calculations using hazard and vulnerability data in 2013 and capacity data for 2021. Thus, the Disaster Risk Index (DRI) values issued illustrate the efforts that have been made to increase capacity so that they can be a guide for policymakers at the national and regional levels to determine the priority of disaster management efforts in their respective regions in order to reduce the DRI as an effort to increase community resilience. In this context, the DRI can be a guide for local governments to prepare disaster mitigation budgets. However, it is rare for previous studies to empirically examine the relationship between the DRI and its use as a budgeting basis by local governments in Indonesia. In this regard, the specific objective is to analyse the empirical relationship between the DRI and the Disaster Management Fund (DMF) in local government in Indonesia. This study contributes practically to stakeholders of local executives in providing a standard budget for disaster mitigation and management in accordance with the DRI. In addition, the results of the study are also expected to be a reference in formulating strategies and budgeting plans that are more accommodating in disaster management in order to reduce the potential impact of socio-economic risks in the future.

## Literature review and hypothesis development

The implementation of disaster management is a series of efforts that include the establishment of development policies that pose a risk of disaster, disaster prevention activities, emergency response and rehabilitation. The DMF is used for all disaster management processes ranging from predisaster, emergency response and/or postdisaster stages. There are some provisions related to the implementation of disaster management in Indonesia. In addition, DMF management was regulated in Law No. 24 of 2007 concerning *Disaster Manageme nt, Government Regulation* No. 22 of 2008 concerning *Funding and Management of Disaster Aid, Regulation of the Head of the National Disaster Management Agency* (Perka BNPB) No. 6 of 2008 concerning *Guidelines for the Use of Ready-to-Use Funds and Perka* BNPB No. 23 of 2010 concerning *Guidelines for Collection and Management of Community Funds for Disaster Management Assistance.*

In this provision, the DMF comes from the state and local government budget and from community participation. The funds originating from state and local government budget are allotted by the central and local governments. The budgets are allotted to disaster contingency funds for preparedness activities at the predisaster stage, ready-to-use funds for activities during emergency response and social assistance funds with a grant pattern for activities in the postdisaster phase in terms of rehabilitation and construction. Meanwhile, related to the management of funds originating from the community, it can be divided into two types. The first is funds received directly by the government or local governments (BNPB or BPBD), whose management follows the grant revenue management mechanism. The second is funds collected and managed directly by community groups such as civic organisations, religious organisations and mass media, although the technical acceptance and distribution are regulated by each of these organisations. The regent or mayor to collect funds from the community is also required to submit a report on the management of the funds with the permission from the Ministry of Social Affairs and the regional governor.

The public budget is a form of planning for government programs and activities that is systematically arranged, expressed in monetary units and determined based on information, assumptions, predictions and agreements of the executive and legislative bodies. Apart from being full of political nuances in its stipulation (Hessami 2014; Lapsley et al. 2011), the level of deviation at the time of its realisation is often used as a performance measurement dimension to describe the effectiveness of public budget policies (Johansson & Siverbo 2014; Zhou et al. 2018). The budgeting of the DMF has a high level of uncertainty and has the potential to have wide deviations or budget insufficiency, especially when disasters occur in large categories and have broad impacts, such as earthquakes, tsunamis and non–natural hazards. Disasters have made drastic changes in the predetermined public budgets and can simultaneously decrease in income and increase in funding in economic and social sectors.

However, Tselios and Tompkins (2020) showed that socio-economic factors, such as state income, level of education, population density, size and governance, have a relationship with the disaster vulnerability of natural and non–natural hazards. A disaster risk mitigation strategy can be in the form of proper planning and budgeting, including increasing investment, economic development, education and governance (Tselios & Tompkins 2020). Likewise, Ward and Shively (2017) found that low-income countries are significantly more at risk of natural hazards, while higher-income countries generally have a lower risk of disaster vulnerability. Postdisaster mortality and morbidity rates in these countries are also lower. These findings suggest that, one strategy that can be taken, is to increase the capacity of disaster management funding. It was also to increase disaster resilience through investment to trigger economic growth and increase government revenues.

Investment can reduce disaster risk (Ishiwata & Yokomatsu 2018). Although disaster risk reduction (DRR) investment costs were found to be more efficient than postdisaster costs, such as costs for emergency response and recovery (Altay, Prasad & Tata 2013), the tendency to invest in disaster prevention or DRR varies widely in different countries (Altay et al. 2013; Keefer, Neumayer & Plümper 2011; Khan et al. 2020; Neumayer, Plümper & Barthel 2014). Harun, An and Kahar (2013), Karim et al. (2020), Abdullah et al. (2020) and Furqan et al. (2020) have provided insights regarding financial management and government budgeting in Indonesia. The results indicate that there are differences in the realisation of local own-source revenue and operational expenditures between provinces in Java and outside Java, which causes differences in the performance of local governments, including the quality of public services provided by local governments. This indicates substantial differences in annual budgeting of disaster management among regions in Indonesia. Previous research findings showed the differences in the budgeting patterns of local governments that have a high DRI compared to those with a lower category of DRI. This ultimately causes differences in the availability of DMFs in each local government. Thus, the hypothesis in this study as detailed in Table 2.

**H1**: There is a positive effect of the Disaster Risk Index on the budgeting of Disaster Management Funds.

**H2**: The Disaster Risk Index has a positive effect on the public service functions in local government.

**TABLE 2:** Descriptive statistics.

Variables	Mean	SD	Min	Max
Disbudg	21.58	1.07	18.42	27.04
RDI	150.19	32.03	44.80	250.00
Elect	0.20	0.40	0.00	1.00
Fiscal	0.12	0.11	0.00	0.84
Size	28.58	0.76	25.19	33.88
Ages	40.49	23.89	1.00	69.00
Island	0.22	0.41	0.00	1.00
Status	0.30	0.57	0.00	2.00

SD, standard deviation.

Number of observations = 2.609.

## Methods

### Data and sample

This research was conducted on local governments in Indonesia. The data used are from 2015–2019 for 542 local governments in Indonesia, with an initial sample of 2710 observations (local government per year). Because there were several observations that did not have complete data, about 101 observations were excluded from the sample. The final sample used in this study was 2609 observations (unbalanced). Secondary data in the study are in the form of the Indonesian DRI document published by the National Disaster Management Agency (BNPB) (2019, 2020); budget data and the value of local government assets from the Ministry of Finance, which are accessed through http://www.djpk.kemenkeu.go.id/?p=5412; data from the General Elections Commission on the implementation of regional election; and data from the Ministry of Home Affairs, especially related to the age of the local government, the status of the local government and the location of the local government.

### Research model and operationalisation of research variables

To answer the research problem, the empirical model in this study is as follows:


$$\begin{array}{l} Disbudg_{it+1}=\alpha_{0}+\alpha_{1}RDI_{it}+\alpha_{2}Elect_{it+1}+\alpha_{3}Fiscal_{it}+\alpha_{4}Size_{it}\\ \;+\alpha_{5}Ages_{it}+\alpha_{6}Island_{it}+\alpha_{7}Status_{it}+\varepsilon_{it} \end{array}$$ [Eqn 1]

*Disbudg*_*it*+1_ is a disaster relief fund allocated by local governments in their annual budget. This was measured by the natural logarithm value of the local government’s annual budget allocated for disaster management emergency response, in the form of the unexpected budget for the following year. *RDI_it_* is a *DRI* variable that describes the level of disaster risk in each province, district and city in Indonesia, which is measured by an index developed from factors in the form of hazard, vulnerability and capacity. *Elect_it_* is a political aspect variable that is measured by dummy regional head elections that occur in the year of the budgeting period, ‘1’ if the following year regional head elections are held and ‘0’ otherwise. *Fiscal_it_* is a variable of fiscal capacity that is measured by the comparison between local revenue and total regional income. *Size_it_* is a local government size variable measured by the natural logarithm value of local government assets. *Ages_it_* is a local government age variable measured by the number of years since the local government was formed. *Island_it_* is a variable for the geographic location of the local government as measured by a dummy, namely ‘1’ for Java and ‘0’ for the rest. *Status_it_* is the status of the area measured categorically, namely ‘2’ for province, ‘1’ for city and ‘0’ for district.

### Ethical considerations

This article followed all ethical standards for research without direct contact with human or animal subjects.

## Results

### Variable overview

Table 2 describes descriptive statistics for all the variables analysed in this study. The mean of the *DRI* variable is 150.19, of which it can be said that the average local government in Indonesia, that is, the research sample, has a *DRI* in the high category. Meanwhile, the mean of 0.12 on the fiscal capacity variable showed that the average local government in Indonesia, which is the sample of this study, still has a high dependence on the central government. The *Ages_it_* variable, which has a mean of 40.49, can be interpreted that the average sample had been formed before the government reform in Indonesia in 1998. As for the *Island_it_* variable and the *Status_it_* variable, the means are 0.22 and 0.30, which means that the average sample used in this study is a local government located outside Java with district status. The results of the correlation analysis between each variable are presented in Table 3.

**TABLE 3:** Correlation analysis.

Variables	Disbudg	RDI	Elect	Fiscal	Size	Ages	Island	Status
Disbudg	1.0000	-	-	-	-	-	-	-
RDI	0.2876** (0.020)	1.000	-	-	-	-	-	-
Elect	−0.0190 (0.324)	−0.045** (0.020)	1.000	-	-	-	-	-
Fiscal	0.4740*** (0.000)	−0.090*** (0.000)	0.005 (0.786)	1.000	-	-	-	-
Size	0.5350*** (0.000)	−0.057*** (0.003)	0.039** (0.046)	0.706*** (0.000)	1.000	-	-	-
Ages	0.2880*** (0.000)	0.094*** (0.000)	0.011 (0.570)	0.364*** (0.000)	0.389*** (0.000)	1.000	-	-
Island	0.2800*** (0.000)	0.102*** (0.000)	−0.005 (0.766)	0.396*** (0.000)	0.343*** (0.000)	0.450*** (0.000)	1.000	-
Status	0.3150*** (0.000)	−0.192*** (0.000)	−0.003 (0.849)	0.628*** (0.000)	0.445*** (0.000)	0.074*** (0.000)	0.036* (0.060)	1.000

Number of observations = 2609.

Explanation of operationalisation of variables in Table 1.

***, **, * , *P*-value significant 1%, 5% and 10%.

Table 3 showed that the DRI and fiscal variables have a positive and significant correlation with disaster relief budgeting *(Disbudg)*. Likewise, with regard to the control variables used, almost all of them are positively and significantly correlated with the disaster relief budgeting. This indicates that disaster response budgeting has a correlation with the DRI, level of fiscal capacity, size, age, location and status of local governments in Indonesia.

### Hypothesis testing

The first specific objective of this study was to analyse the relationship between the DRI and the availability of DMFs in local governments in Indonesia. Based on these objectives, the first hypothesis of this study is suspected to have an influence on the DRI on the budgeting of DMFs. To test this hypothesis, the ordinary least square test is used using the Stata 14 program (StataCorp LLC, College Station, Texas, United States). The results of testing Hypothesis 1 are presented in Table 4.

**TABLE 4:** The effect of the Disaster Risk Index on Disaster Management Funds.

Variable	Expected sign	Coef. (*P*)
(1)	(2)	(3)
_CONS	-	5.765 (0.000)
RDI	(+)	0.001** (0.011)
Elect	(±)	−0.086 (0.117)
Fiscal	(+)	1.037*** (0.000)
Size	(+)	0.533*** (0.000)
Ages	(?)	0.002*** (0.002)
Island	(?)	0.184*** (0.000)
Status	(?)	0.147*** (0.000)
Prob > chi^2^	-	0.000
Adj. *R*-squared	-	31.930
Obs.	-	2.609

Variable *Y* = Disbudg.

Explanation of operationalisation of variables in Table 1.

***, **, * , *P*-value significant 1%, 5% and 10%.

Table 4 presents the results of testing the factors that influence the budgeting of DMFs in local governments in Indonesia. In general, the results of the model test show an adjusted *R*-squared value of 31.93%, significant at the 1% level. These results mean that 31.93 variations in budgeting for DMFs in local governments can be explained by the variables studied in this study. The results of hypothesis testing in Table 3 show that the DRI variable has a positive effect on the Disbudg variable with a coefficient of 0.001, significant at the 5% level. Thus, the data used in this study support the first hypothesis. This means that the DRI influences the disaster emergency budgeting in Indonesia local governments.

Likewise, the Fiscal variable has a positive effect on the Disbudg variable with a coefficient of 1.037, significant at the 1% level, so the findings showed that the fiscal capacity of a region has been used as the basis for budgeting for DMFs in local governments in Indonesia. Meanwhile, the Elect variable has no significant effect on the Disbudg variable. This means there is no influence of political factors on the budgeting of DMFs in local governments in Indonesia.

Table 3 also showed that the variables Size, Ages, Island and Status have a positive effect on the Elect variable with coefficients of 0.533, 0.002, 0.184 and 1.037, respectively, significant at the 1% level. This can be interpreted that the emergency response fund budgeting at the local government level, which has a large asset value, has long been formed, is located on the island of Java and has a provincial status is greater than the emergency response fund budgeting at the local government with low asset values, newly formed, outside the island of Java and the status of regency or city.

Furthermore, the second specific objective of this study was to analyse the relationship between the pattern of budgeting for the implementation of regional functions and the DRI. This is the basis for Hypothesis 2: it is suspected that there is an influence of the DRI on the budgeting of the implementation of local government functions. To test Hypothesis 2, the test is carried out on the budget for functions related to public services (Service variable in column 2), housing and public facilities (Facility variable in column 3), environment (Environment variable in column 4), health (Health variable in column 5), economy (Economy variable in column 6) and social function (Social variable in column 7). The results of testing hypothesis 2 are presented in Table 5.

**TABLE 5:** The effect of the Disaster Risk Index on public service functions of local governments.

Variables	Coef. (*P*)					
	Service	Facility	Environment	Health	Economy	Social
(1)	(2)	(3)	(4)	(5)	(6)	(7)
_CONS	13.228 (0.000)	12.067 (0.000)	13.971 (0.000)	15.532 (0.000)	12.180 (0.000)	13.527 (0.000)
RDI	0.002*** (0.000)	0.001*** (0.000)	−0.001* (0.052)	0.001*** (0.000)	0.001 (0.220)	0.000 (0.139)
Elect	0.063*** (0.001)	−0.176** (0.024)	−0.038** (0.014)	0.064* (0.083)	−0.303* (0.099)	−0.005 (0.891)
Fiscal	0.653** (0.014)	0.911*** (0.000)	2.217*** (0.000)	1.334*** (0.000)	0.647*** (0.006)	0.769*** (0.000)
Size	0.463*** (0.000)	0.481*** (0.000)	0.326*** (0.002)	0.349*** (0.000)	0.457*** (0.000)	0.352*** (0.000)
Ages	0.006*** (0.000)	−0.001*** (0.002)	0.001** (0.045)	0.006*** (0.002)	0.001*** (0.000)	0.001 (0.596)
Island	−0.002 (0.960)	−0.089 (0.149)	0.365*** (0.149)	0.208*** (0.000)	0.065 (0.481)	0.021 (0.673)
Status	−0.017 (0.615)	−0.023 (0.441)	−0.016 (0.582)	−0.127*** (0.000)	0.098* (0.086)	0.106*** (0.000)
Prob > chi^2^	0.000	0.000	0.000	0.000	0.000	0.000
Adj. *R*-squared	31.960	34.870	32.310	51.160	38.250	32.690
Obs.	2.609	2.609	2.609	2.609	2.609	2.609

Variable *Y* = Budgeting for the implementation of local government functions, which consists of Service (column 2), Facility (column 3), Environment (column 4), Health (column 5), Economy (column 6) and Social (column 7).

The budgeting variable for the implementation of local government functions is a variable for budgeting the implementation of local government functions as measured by the natural logarithm value of the local government budget allocated to carry out each of the functions of public services, housing and public facilities, environment, health, economy and social functions.

Explanation of the operationalisation of other variables in Table 1.

***, **, * , *P*-value significant 1%, 5% and 10%.

Table 5 generally presents the results of testing the second hypothesis. The findings found that all models are significant at the 1% level, with adjusted *R*-squared between 32.31% and 51.16%. The findings also demonstrated that the second hypothesis is only proven in budgeting for the function of public services, public facilities and health spending. The findings showed that the DRI has not been fully used as the basis for disaster management; it is still limited to the stage of disaster emergency response services, namely the functions of public services, public facilities and health. Meanwhile, for indirect impacts, such as social and economic problems that are likely to have an impact because of disasters, the budgeting has not been based on the DRI. To mitigate the risk of natural hazards, such as floods and landslides, which was originally carried out by strengthening the quality of the environment, the DRI was found to have a negative effect.

### Sensitivity testing

The Disaster Management Agency has categorised the DRI in Indonesia into three categories, namely high (DRI score > 144), medium (DRI score 13–144) and low (DRI score < 13). As explained in Table 1 (variable descriptive statistics), the regional DRI scores during the observation period in this study were in the range of 44.8 – 250, or they consisted of high and medium categories. Table 3 showed a sensitivity test was carried out using the DRI variable as measured by the DRI category dummy, namely ‘1’ for high-category DRI scores and ‘0’ for other categories. The results of the sensitivity test are presented in Table 6.

**TABLE 6:** Sensitivity test results.

Variable	Expected sign	Coef. (*P*)
(1)	(2)	(3)
_CONS	-	6.090 (0.000)
RDIdummy	(+)	0.073* (0.058)
Elect	(±)	−0.089* (0.099)
Fiscal	(+)	1.027*** (0.000)
Size	(+)	0.530*** (0.000)
Ages	(?)	0.002*** (0.001)
Island	(?)	0.194*** (0.000)
Status	(?)	0.137*** (0.000)
Prob > chi^2^	-	0.000
Adj. *R*-squared	-	31.77
Obs.	-	2.609

Variable *Y* = Disbudg.

RDI_dummy_ is a disaster risk index (DRI) variable that describes the level of disaster risk in each province, regency and city in Indonesia, which is measured by a dummy for the DRI category, namely ‘1’ if the category is high and ‘0’ otherwise.

Explanation of the operationalisation of other variables in Table 1.

***, **, * , *P*-value significant 1%, 5% and 10%.

Table 6 generally showed that the variables of this research model can explain 31.77% of the variation in budgeting for DMF, significant at the 1% level. The variable of the DRI has a positive effect on the Disbudg variable with a coefficient of 0.073, which is significant at the 10% level. The results of sensitivity testing for other variables show that the DRI has been used as the basis for budgeting for DMF in local governments in Indonesia, and it is robust with various DRI measurements, both using the DRI score and the DRI category.

## Conclusion

This study aimed to develop a local government budgeting model based on the DRI, in particular to analyse the influence of the DRI on the budgeting of DMFs and the implementation of local government functions. This study used a sample of local governments in Indonesia consisting of province, regency and municipality, especially for 2015–2019 data with a final sample of 2609 observations.

The results of the analysis and testing show that during the year of observation, the majority of regions in Indonesia had a DRI in the high category with an average DRI score of 150.19. This certainly showed that the disaster resilience in Indonesia is still very low. Strengthening disaster resilience and risk management must receive more serious attention in budgeting for regional disaster management. The results also show that the DRI significantly affects the budgeting of regional emergency response funds. This finding is robust to the differences in DRI measurements, both using scores and DRI categories. This study also found that the DRI has been used as the basis for budgeting regional expenditures, particularly in relation to the budget for the implementation of public service functions, housing and public facilities and public health. Meanwhile, budgeting for the implementation of economic and social functions is not influenced by the DRI; the DRI was even found to have a negative effect on the implementation of environmental functions.

In general, the findings showed that the DRI has been used as the basis for budgeting for regional disaster management. Nonetheless, it is still limited to functions related to disaster emergency response. Meanwhile, budgeting for the implementation of preventive activities has not been optimally carried out, especially mitigating natural hazards through strengthening the quality of the environment. The results are expected to contribute to the local government in order to improve local disaster resilience through strengthening regional financial funding.

One of the limitations of this study that needs attention in further research is the limited data in describing the entire budget managed by the Regional Disaster Management Agency (BPB). It is less likely to explain in detail how much the local government has prepared for all disaster management activities, starting from the preventive activities and mitigation, through the emergency response to the postdisaster recovery in each region, especially those managed by BPBD as the leading sector in regional disaster management. It is thus recommended for further research to analyse the impact of the realisation of the disaster management funding budget on disaster resilience or DRI in each region in the following years.
